# A Case of Hybrid Stent Placement Following Bronchial Artery Embolization for Bleeding Airway Stenosis due to Mediastinal and Hilar Lymph Node Metastasis by Renal Cell Carcinoma

**DOI:** 10.1002/ccr3.73239

**Published:** 2026-07-28

**Authors:** Yuto Sasano, Yuki Takigawa, Ken Sato, Mayu Uka, Satoshi Nogami, Shoichiro Matsumoto, Tomoyoshi Inoue, Suzuka Matsuoka, Hiromi Watanabe, Hiroe Kayatani, Kenichiro Kudo, Keiichi Fujiwara, Takuo Shibayama

**Affiliations:** ^1^ Department of Respiratory Medicine NHO Okayama Medical Center Okayama Japan; ^2^ Department of Radiology NHO Okayama Medical Center Okayama Japan; ^3^ Department of Anesthesiology NHO Okayama Medical Center Okayama Japan; ^4^ Department of Respiratory Medicine Japanese Red Cross Okayama Hospital Okayama Japan

**Keywords:** AERO stent, airway stenosis, bronchial arterial embolization, renal cell carcinoma

## Abstract

Here, we report a rare case of severe central airway obstruction caused by mediastinal and hilar lymph node metastases with persistent bleeding from renal cell carcinoma. Hemoptysis and critical left main bronchial stenosis were initially managed with bronchial arterial embolization, which reduced the risk of bleeding during subsequent interventions. This enabled safe placement of an AERO airway stent, leading to a rapid improvement in respiratory status. The patient was able to proceed with systemic therapy without delay. This case demonstrates the value of combining bronchial arterial embolization (BAE) with airway stenting as an effective bridge strategy for the treatment of life‐threatening malignant airway stenoses.

**Key Clinical Message**

Airway stenosis caused by renal cell carcinoma metastases is frequently associated with bleeding and the risk of life‐threatening airway obstruction. Inadequate or delayed intervention can result in fatal outcomes. Bronchial artery embolization combined with guide wire‐assisted placement of an AERO stent should be considered for airway patency and control bleeding.

## 1 Introduction

Airway stenosis due to hilar and mediastinal lymph node metastases of renal cell carcinoma (RCC) is rare. Because RCC is a highly vascular tumor, endobronchial or intrapulmonary metastases may cause hemoptysis and carry a high risk of asphyxiation. Although chemotherapy and radiotherapy are generally used for treatment, airway stenting can provide rapid relief in cases of airway stenosis [1], as it restores airway patency and improves respiratory status, activities of daily living (ADL), and quality of life (QOL) before definitive therapies are effective. Bronchial arterial embolization (BAE) is another effective modality for managing hemoptysis and airway obstruction. Rigid bronchoscopic intervention for metastasis of renal cell carcinoma requires sufficient preparation for massive intraoperative bleeding for safer treatment [2]. We report a case in which BAE followed by airway hybrid stent placement successfully improved the outcome of a patient with airway stenosis caused by pulmonary metastasis of RCC.

## 2 Case History/Examination

A 63‐year‐old man presented with hemoptysis and dyspnea. His medical history included left‐sided RCC. The patient had no history of anticoagulant or antiplatelet use. He had a smoking history of 30 pack‐years, and he was independent in ADLs. In April 2024, the patient developed a cough, and computed tomography (CT) revealed lymphadenopathy and a left renal mass. A biopsy of a hilar lymph node confirmed RCC with hilar and mediastinal lymph node metastases at a previous hospital. The initiation of anti‐cancer therapy was delayed for patient‐related reasons. Two months after diagnosis, hemoptysis and dyspnea worsened due to the enlargement of the mediastinal and left hilar lymph node with bronchial invasion causing airway stenosis, and he was transferred to our hospital for pulmonary intervention.

## 3 Differential Diagnosis, Investigations and Treatment

On admission, the patient was afebrile with stable vital signs except for an oxygen requirement of 3–5 L/min (SpO_2_ 92%). He continued to experience hemoptysis of approximately 20 mL/day. Arterial blood gas analysis showed a PaO_2_ of 86 mmHg while receiving oxygen at 3 L/min. The Eastern Cooperative Oncology Group Performance Status (ECOG PS) was three.

**FIGURE 1:**
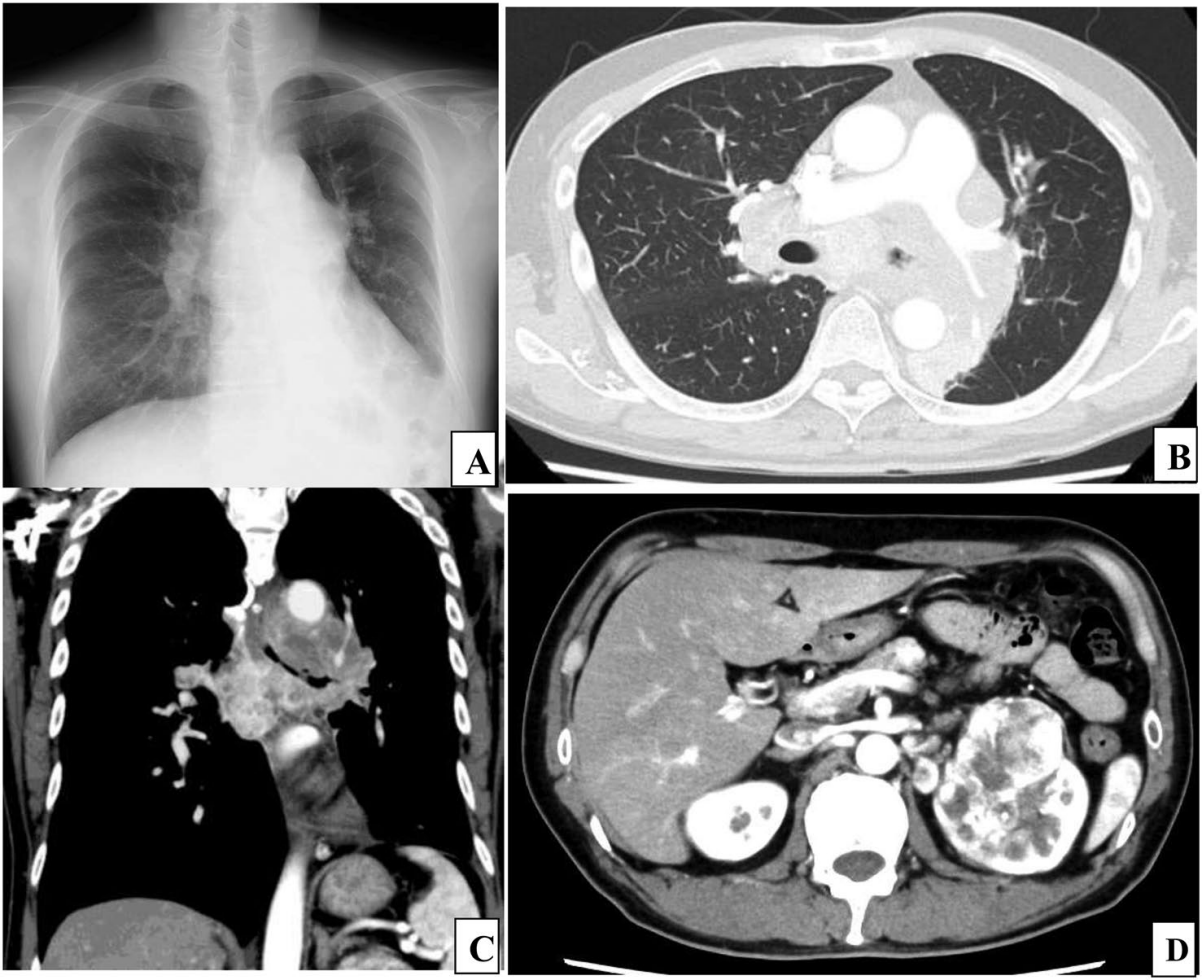
(A) Chest radiograph shows atelectasis in the left lower lung field. (B) Lung window. (C) Mediastinal window. Chest contrast‐enhanced CT revealed enlarged mediastinal lymph nodes with internal necrosis, causing compressive and infiltrative narrowing of the left main bronchus. (D) Abdominal enhanced CT shows renal cell carcinoma with internal necrosis and early dark staining at the upper pole of the left kidney.

Chest radiography revealed left lower lobe atelectasis (Figure 1A). Contrast‐enhanced CT revealed necrotic mediastinal lymphadenopathy causing severe compression and invasion of the left main bronchus (Figure 1B,C), along with a 7‐cm upper renal mass consistent with RCC (Figure 1D).

**FIGURE 2:**
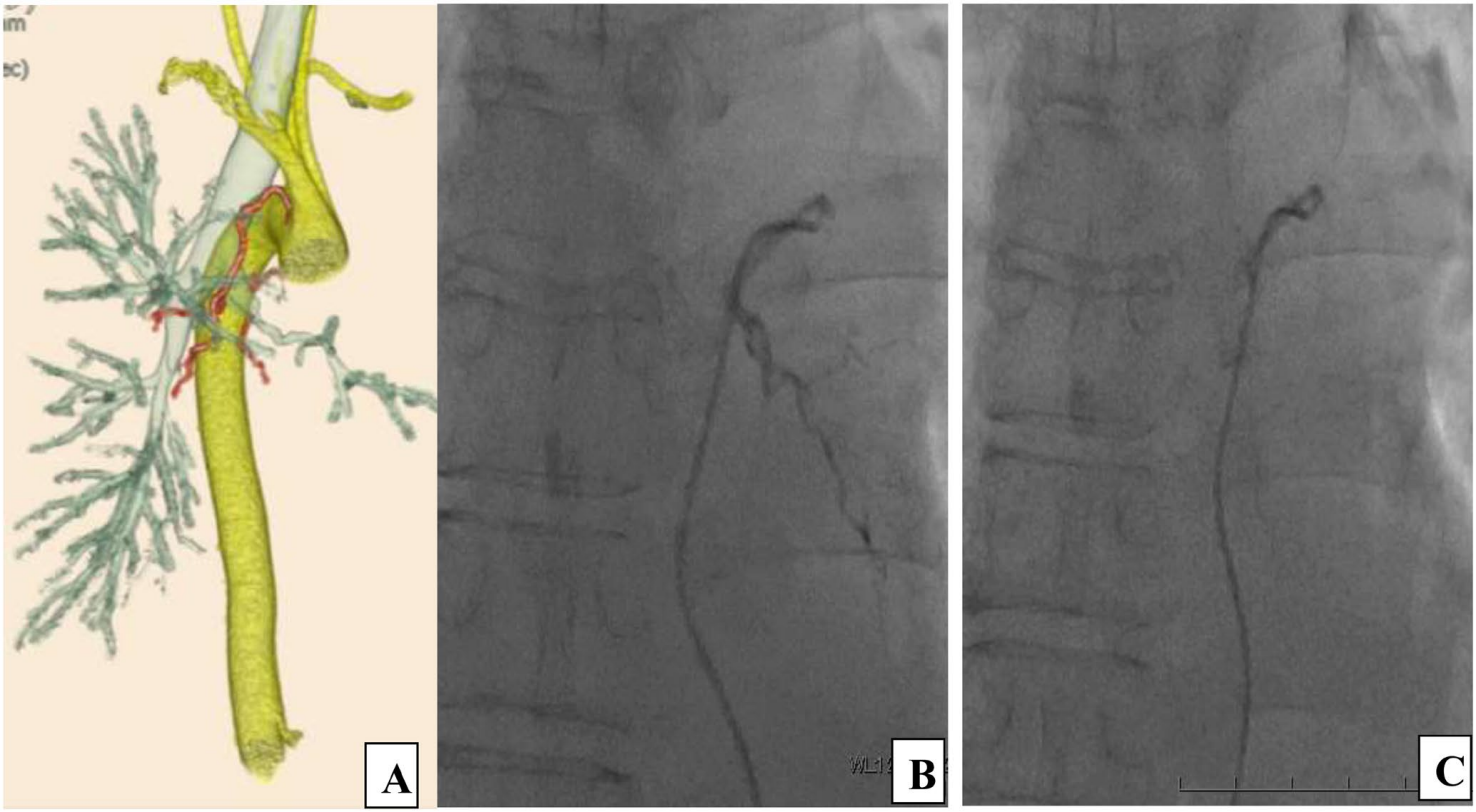
Three dimensional CT image shows an aberrant artery branching from the brachiocephalic artery of the right bronchial artery. The gelatin sponge embolized the left bronchial artery. Bronchial angiography after BAE reveals disappearance of vascular shadows. (A) Angiography image. (B) Before BAE. (C) After BAE.

On hospital day 1, BAE was performed by an interventional radiologist. An aberrant right bronchial artery arising from a site immediately adjacent to the origin of the brachiocephalic artery was identified (Figure 2A). Because embolization of the right bronchial artery may carry a risk of cerebral infarction, only the left bronchial artery was embolized using a gelatin sponge (Figure 2B,C). Post‐embolization angiography confirmed elimination of the target vessel, and the hemoptysis gradually improved. Although the volume of hemoptysis decreased, minor hemoptysis persisted, and airway patency needed to be secured using a stent.

**FIGURE 3:**
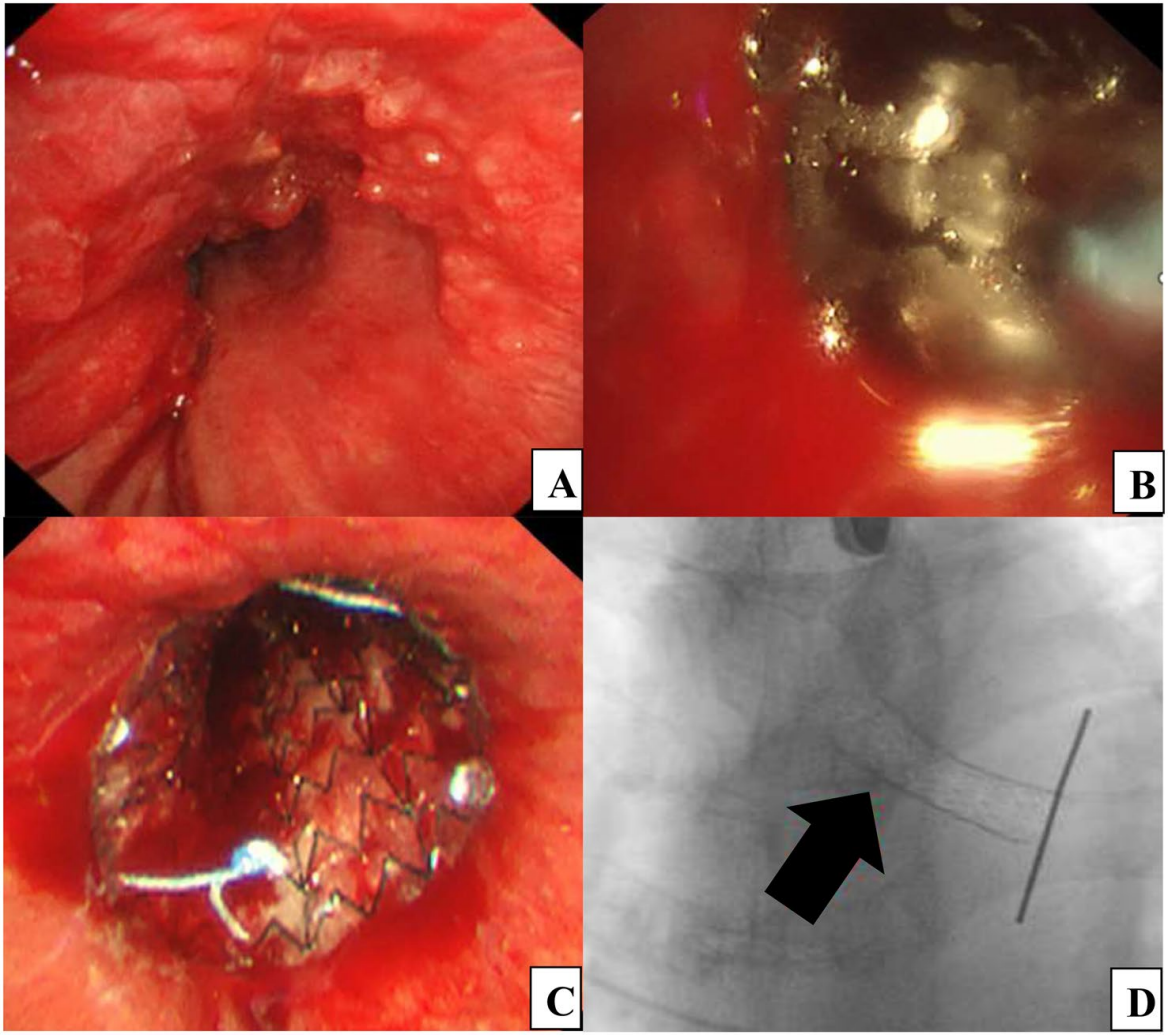
(A) The left main bronchus is 90% stenosed, with oozing from the elevated lesion. (B) Balloon dilatation of left main bronchus with 10‐11‐12 mm 3 step balloon. (C) Placement of AERO Stent. (D) Black arrow shows placement of AERO Stent under fluoroscopy.

On hospital day 4, an airway intervention via rigid bronchoscopy was performed under general anesthesia. A flexible bronchoscope (BF‐P290 and 1TQ290; Olympus, Japan) was inserted via rigid bronchoscope intubation. Bronchoscopy revealed severe stenosis of the left main bronchus, with approximately 90% luminal narrowing (Grade 4, according to a previously reported [3] classification) and minimal bleeding (Figure 3A). Balloon dilation was performed using a 10‐11‐12 mm CRE balloon (Boston Scientific, US) (Figure 3B), followed by placement of a bronchial hybrid stent (AERO stent, Merit Medical, US; 12 × 40 mm) using the over‐the‐wire technique (Figure 3C,D). After stent deployment, Argon plasma coagulation (1.5 mm FiAPC‐probe; Erbe, Germany) was applied to a small bleeding site that was not covered by the stent.

## 4 Conclusion and Results (Outcome and Follow‐Up)

**FIGURE 4:**
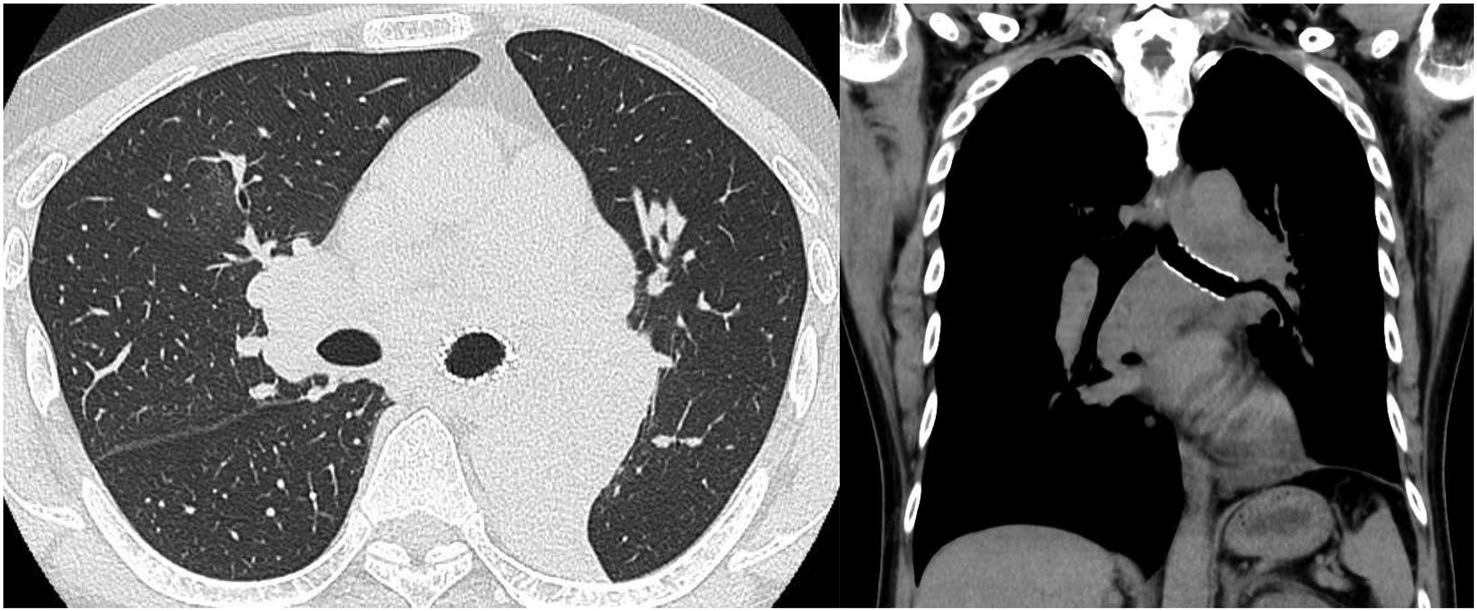
CT finding after AERO stent placement.

Hemoptysis was resolved on hospital day 5, and the oxygen requirements decreased. CT confirmed appropriate stent position and airway patency (Figure 4). The patient was transferred back to the referring hospital on day 7 to begin anti‐cancer therapy.

## 5 Discussion

Herein, we present a case in which preprocedural BAE enabled safe stent placement for airway stenosis caused by endobronchial bleeding from metastatic RCC. Although the lungs are a common site of RCC metastasis owing to their rich vascularity [4], metastasis to the bronchi or mediastinal lymph nodes is relatively uncommon. Previous retrospective studies have shown that BAE reduces severe bleeding risk and enables safer endoscopic procedures in metastatic RCC [5] Our previous retrospective study suggested that preoperative BAE may reduce the risk of bleeding during subsequent airway stent placement in patients with bleeding malignant airway stenosis [6]. In addition, this approach may facilitate hemostasis, and any improvement in airway stenosis may have been partly attributable to a reduction in tumor burden following embolization [7]. However, BAE carries potential complications such as chest pain, bronchial wall necrosis, pulmonary infarction, spinal cord infarction, and dysarthria, requiring careful monitoring [8].

The AERO stent is a hybrid bronchial stent characterized by its self‐expanding properties, fully covered design, and ease of removal. Unlike silicone stents, which often require the push‐out or pull‐back method during placement and may cause friction‐related airway mucosal injury, the self‐expanding power of the AERO stent allows for deployment with minimal airway damage. Compared to silicone stents, it offers superior conformability and adaptability to stenotic characteristics, and its self‐expanding force is expected to provide a high hemostatic effect. In addition, the AERO stent can also be removed or repositioned relatively easily under a rigid bronchoscope, making it an attractive option for malignant airway stenosis, in which future stent removal is considered. Previous reports have shown that the AERO stent is effective as a bridging therapy even in critical patients or those with poor performance status, and that subsequent anti‐cancer therapy after stent placement may contribute to prolonged survival [9, 10]. In the present case, systemic therapy was planned following stent placement, and improvement of airway obstruction through tumor reduction was expected. Similar case reports are extremely limited, and cases in which both BAE and airway stent placement are combined as therapeutic interventions for airway obstruction caused by metastatic RCC are rare.

Airway stenosis caused by RCC metastases is frequently associated with bleeding and carries the risk of life‐threatening airway obstruction. Inadequate or delayed intervention may lead to fatal outcomes during treatment. As a potential therapeutic strategy, BAE combined with guide wire‐assisted placement of an AERO stent should be considered in order to achieve airway patency and control bleeding.

## Author Contributions

**Yuto Sasano:** conceptualization, writing – original draft. **Yuki Takigawa:** conceptualization, supervision, writing – review and editing. **Ken Sato:** supervision, writing – review and editing. **Mayu Uka:** conceptualization, visualization. **Satoshi Nogami:** conceptualization, visualization. **Shoichiro Matsumoto:** conceptualization, visualization. **Tomoyoshi Inoue:** conceptualization, visualization. **Suzuka Matsuoka:** conceptualization, visualization. **Hiromi Watanabe:** conceptualization, visualization. **Hiroe Kayatani:** conceptualization, visualization. **Kenichiro Kudo:** conceptualization, visualization. **Keiichi Fujiwara:** data curation, visualization. **Takuo Shibayama:** conceptualization, visualization.

## Funding

The authors have nothing to report.

## Ethics Statement

The authors have nothing to report.

## Consent

Written informed consent was obtained from the patient for the publication of this case report and any accompanying images.

## Conflicts of Interest

The authors declare no conflicts of interest.

## Data Availability

All data generated or analyzed during this study are included in this published article.
